# Simultaneous ipsilateral fractures of distal and proximal ends of the radius

**DOI:** 10.11604/pamj.2017.27.98.3504

**Published:** 2017-06-08

**Authors:** Khalid Ibn El Kadi, Mounir Benabid, Sarr Saliou, Oussama El Assil, Amine Marzouki, Kamal Lahrach, Fawzi Boutayeb

**Affiliations:** 1 Department of Orthopedic Surgery (A), UH Hassan II, Fes, Morocco

**Keywords:** Radius, ipsilateral, radial head, distal radial fractures

## Abstract

We treated a patient with a rare combination of ipsilateral fractures of the distal and proximal ends of the radius. A man aged 42 years had simultaneous fractures of the distal and proximal ends of the radius (radial neck) following a roadside accident. The distal end fracture of the radius was treated with surgical reduction and T-plate volar fixation, and the undisplaced radial neck fracture was treated by an above elbow splintage for 2 weeks. The elbow mobilization was started at 2 weeks. The distal radius was protected for another 4 weeks in a below elbow functional brace. Ipsilateral proximal and distal radial fracture is an uncommon injury pattern. The series illustrates a number of problems associated with this combination. Firstly, one should be aware of this rare injury pattern and there should be greater emphasis on clinical examination of elbow in cases of wrist injuries and vice versa. Once diagnosed, one faces the dilemma of appropriate management in these cases. The appropriate management will depend on the injury characteristics including the age of the patient and the fracture pattern. One should try to preserve the radial head to prevent a possible proximal radial migration especially in younger patients.

## Introduction

Fractures of the distal end of the radius are commonly encountered in clinical practice, while fractures of the proximal end of the radius occur mostly when an individual falls with the impact on the outstretched hand, with the elbow joint extended; these fractures should be treated with special attention to associated injury of the ulnar collateral ligament [1]. However, Only few references in indexed literature make a mention of bifocal fractures of radius [2, 3]. We report a rare case of ipsilateral fractures of the distal and proximal ends of the radius, and we discuss the mechanism of these fractures and their treatment in this patient.

## Patient and observation

**Figure 1:**
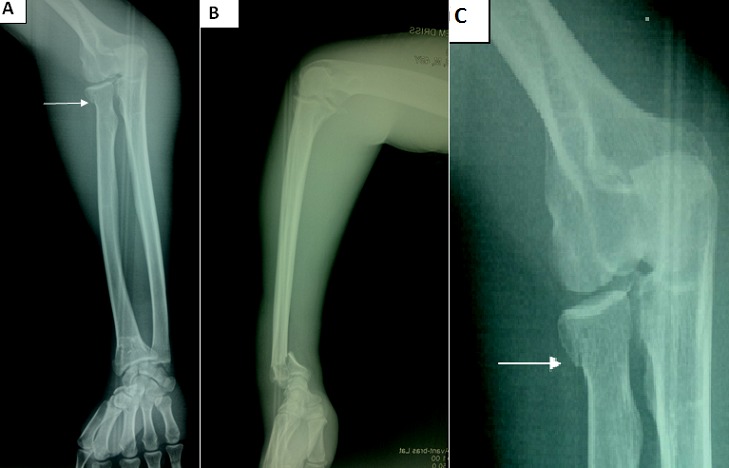
Preoperative radiograph of the right forearm, showing a combination of fractures of the distal and proximal ends of the radius (A, B); the undisplaced radial neck fracture (arrow) (C)

**Figure 2:**
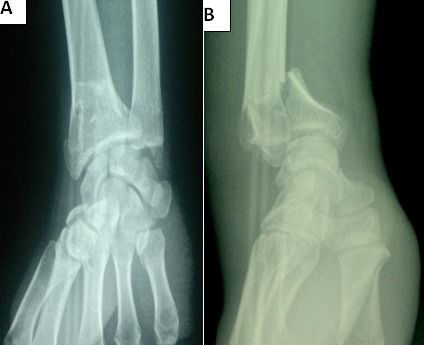
(A) preoperative anteroposterior radiograph of the left wrist joint, showing an intra-articular fracture of distal radius; (B) preoperative lateral radiograph of the right wrist joint, showing a volar displacement: Goyrand-smith fracture with sagittal articular fracture line

**Figure 3:**
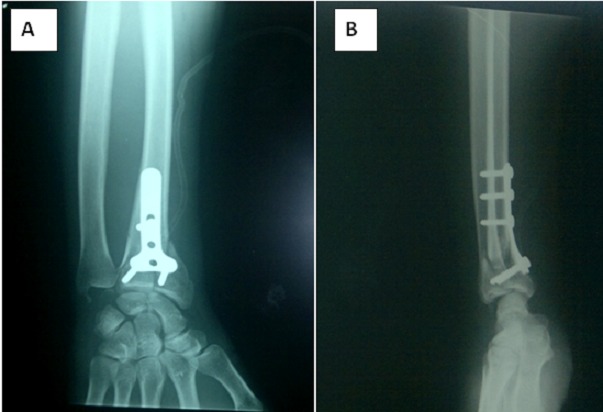
(A) anteroposterior radiograph of the left wrist joint immediately after surgery: the fractured distal end of the radius fixed with a T-volar plate fixation; (B) postoperative lateral radiograph of the left wrist

A man aged 42 years presented with simultaneous fractures of the distal and proximal ends of the left radius (radial neck) (Figure 1) after a roadside accident (fall of a motorcycle). The fracture of the distal end of the radius was found to be a Goyrand- smith with sagittal articular fracture line (Classification of Castaing and le Club des Dix [4]) (Figure 2), while the fracture of the proximal end of the radius was a undisplaced radial neck fracture (Mason type I fracture) [5]. Physical examination showed swellingand ecchymosis in the radial region of the elbow joint, and pain with wrist strain. Neurovascular examination of the upper limb was normal. The patient was surgically treated, under locoregional anesthesia of the upper limb (Brachial plexus block ), by open reduction of the fractured distal end of the radius. A dissection was made between the flexor carpi radialis and palmaris longustendons. The flexor pollicis longus tendon was retracted in the direction of the radius, while the median nerve and other tendons were retracted in the direction of the ulna, revealing the pronator quadratus. Next, the distal and radial borders of the pronator quadratus were raised and retracted in the direction of the ulna to expose the distal radius. Next, open reduction of the distal radius was performed with the aid of intrafocal leverage achieved via elevation, traction, and fixation using temporary Kirschner wires, Finally, the distal radius fracture was fixed with a T-volar plate fixation (Figure 3). The undisplaced fractured radial neck was treated by an above elbow splintage for 2 weeks. The elbow mobilization was started at 2 weeks. The distal radius was protected for another 4 weeks in a below elbow functional brace. Three months after the surgery, recovery of range-of-joint motion (elbow joint, 135° flexion and -5° extension; forearm, 80° supination and 50° pronation; wrist joint 80° dorsal flexion and 70° palmar flexion) was present, without complaint of pain on movement or detectable loss of grip strength (right, 38 kg; left, 36kg), and the patient had a good result (90 points) based on Cooney's score [6] (pain, no pain; functional status, returned to regular employment; range of motion, 75%-100%; grip strength, 100%).

## Discussion

Fractures of the distal end of the radius are often associated with elbow joint dislocation, but rarely with fractures of the proximal end of the radius. Although the reason for this finding is not clear, a fracture of the distal end of the radius may reduce axial pressure applied to the radius, and, thereby, reduce the possibility of an additional fracture occurring at the proximal end of the same radius. Both fractures of proximal and distal radius are quite common individually in adults and children. In fact, distal radius fractures account for 14% of all extremity injuries and 17% of all adult fractures treated in emergency departments [7, 8]. Similarly, the radial head and neck fracture is another common adult injury. The concomitant occurrence of ipsilateral fractures in proximal and distal radius with or without associated ulna/olecranon fracture is a relatively rare phenomenon. Nagaya et al. [1] described one such case in a 52-year female. The fracture of the radial head was treated by open fixation with a cancellous bone screw and distal end radius by application of external fixator and bone grafting. A similar injury in children was referred by Waters [3] Bado [9] and many other authors have described occurrence of distal radial fractures in conjunction with Monteggia lesions or equivalents/various combinations [10]. Agarwal described another series of five cases with fractures of either radial end with or without an associated ulnar fracture and their outcome. Two of these patients belong to pediatric age group, thereby indicating that no age group is exempt from this fracture pattern [8]. The exact series of events which resulted in this particular injury pattern is difficult to speculate. There are several possible mechanisms which can produce a bifocal lesion of radius and in some cases, an associated fracture of proximal ulna as well. The mechanisms will depend on whether the elbow is flexed or extended and the position of the forearm at the time of injury. If the elbow is flexed, posterior tension forces play an important role [11]. If an associated abduction thrust is present, a compression force is created across the radiocapitellar joint. The proximal radius fractures as a result of radiocapitellar impaction (our patient). Henrikson found that persons with increased carrying angle are predisposed to valgus stress in falls with elbow extended, resulting in fracture of proximal radius and/ulna [12]. The direction of fracture angulation depends on whether the forearm is in supination, neutral or pronated position at the time of fall.The injury to the medial aspect of elbow may either be pure ligamentous or there may be fracture of proximal ulna. The elbow may temporarily dislocate at the time of impact . Similarly, a varus strain can produce fracture of proximal radius and ulna [13].

A less common explanation for these types of injuries may be a direct blow producing a fracture at one radial site along with an axial force created by fall [11]. Looking to the fracture comminution and the associated fractures and after maths, it appears that there is a strong axial component in the forces causing this double injury. In older age group, especially elderly women, the fracture pattern can be a result of low energy trauma with minimal displacement of fractures and possibly an intact interosseous membrane and more likelihood of good results. In younger age group, the injury complex results from high-energy trauma with comminution, instability and significant soft tissue damage. The energy of trauma and skeletal maturity probably dictates the prognosis in these injuries [8]. The proximal injury of this fracture complex share similarities with several named fractures of this region: Monteggia equivalent fractures: elaborated by Bado [9], these embrace several variants of Monteggia fracture including a radial neck fracture. The association of olecranon/proximal ulnar fracture along with proximal radial injury may represent an extension of these ''equivalent lesions''. Essex Lopresti fracture: an injury involving fracture of radial head and neck, disruption of the distal radioulnar joint and interosseous membrane [14]. In such cases, if the radial head is resected, there is propensity to develop wrist pain from ulnar carpal impingement and elbow pain from radiocapitellar impingement.This study ,shows, the importance of complete clinical and radiological examination of elbow in all cases of wrist fracture and vice versa. An occult radial fracture may be initially overlooked because of more obvious pain and deformity of a distal radius fracture. In a child, even with a proximal radial fracture, the primary complaint may be only referred wrist pain [2]. Persons with increased carrying angle are prone to valgus stress at elbow in extended elbow position leading to radiocapitellar impaction [12]. Hence, a systematic examination of the elbow after a distal radial fracture in patients with increased carrying angle is also emphasized. It is advocated to include radiographs of elbow joint in all cases of distal radius fractures where there is even slightest suspicion of a concomitant elbow trauma. The treatment of this double injury depends upon fracture characteristics of proximal and distal fracture. Clearly the fracture pattern, the degree of displacement, the stability of the fracture, the age and the physical demands of the patient determine the best treatment option. We suggest that a more conservative attitude of radial head preservation be adopted to prevent a proximal radial migration in view of a possible associated interosseous membrane injury especially in younger patients. Surgical intervention should be opted where an acceptable reduction cannot be achieved or maintained by close means. The appropriate diagnostic and therapeutic management of our patient explains the good functional recovery of elbow and wrist. In the paediatric group, the fracture pattern was complicated by avascular necrosis of radial epiphysis and premature physeal fusion [11, 13, 15].

## Conclusion

Ipsilateral proximal and distal radial fracture is an uncommon injury pattern. The series illustrates a number of problems associated with this combination. Firstly, one should be aware of this rare injury pattern and there should be greater emphasis on clinical examination of elbow in cases of wrist injuries and vice versa. Once diagnosed, one faces the dilemma of appropriate management in these cases. The appropriate management will depend on the injury characteristics including the age of the patient and the fracture pattern. One should try to preserve the radial head to prevent a possible proximal radial migration especially in younger patients.

## Competing interests

The authors declare no competing interests.
